# Image quality characterization of an ultra‐high‐speed kilovoltage cone‐beam computed tomography imaging system on an O‐ring linear accelerator

**DOI:** 10.1002/acm2.14337

**Published:** 2024-04-04

**Authors:** Euidam Kim, Yang Kyun Park, Tianyu Zhao, Eric Laugeman, Xiaodong Neo Zhao, Yao Hao, Yoonsun Chung, Hugh Lee

**Affiliations:** ^1^ Department of Radiation Oncology Washington University in St Louis School of Medicine St Louis Missouri USA; ^2^ Department of Nuclear Engineering Hanyang University College of Engineering Seoul South Korea; ^3^ Department of Radiation Oncology University of Texas Southwestern Medical Center Dallas Texas USA

**Keywords:** cone‐beam computational tomography (CBCT), image characterization, image quality, imaging quality assurance (QA)

## Abstract

**Purpose:**

The quality of on‐board imaging systems, including cone‐beam computed tomography (CBCT), plays a vital role in image‐guided radiation therapy (IGRT) and adaptive radiotherapy. Recently, there has been an upgrade of the CBCT systems fused in the O‐ring linear accelerators called HyperSight, featuring a high imaging performance. As the characterization of a new imaging system is essential, we evaluated the image quality of the HyperSight system by comparing it with Halcyon 3.0 CBCT and providing benchmark data for routine imaging quality assurance.

**Methods:**

The HyperSight features ultra‐fast scan time, a larger kilovoltage (kV) detector, a more substantial kV tube, and an advanced reconstruction algorithm. Imaging protocols in the two modes of operation, treatment mode with IGRT and the CBCT for planning (CBCTp) mode were evaluated and compared with Halcyon 3.0 CBCT. Image quality metrics, including spatial resolution, contrast resolution, uniformity, noise, computed tomography (CT) number linearity, and calibration error, were assessed using a Catphan and an electron density phantom and analyzed with TotalQA software.

**Results:**

HyperSight demonstrated substantial improvements in contrast‐to‐noise ratio and noise in both IGRT and CBCTp modes compared to Halcyon 3.0 CBCT. CT number calibration error of HyperSight CBCTp mode (1.06%) closely matches that of a full CT scanner (0.72%), making it suitable for adaptive planning. In addition, the advanced hardware of HyperSight, such as ultra‐fast scan time (5.9 s) or 2.5 times larger heat unit capacity, enhanced the clinical efficiency in our experience.

**Conclusions:**

HyperSight represented a significant advancement in CBCT imaging. With its image quality, CT number accuracy, and ultra‐fast scans, HyperSight has a potential to transform patient care and treatment outcomes. The enhanced scan speed and image quality of HyperSight are expected to significantly improve the quality and efficiency of treatment, particularly benefiting patients.

## 1 INTRODUCTION

Image‐guided radiation therapy (IGRT) has been a mainstream radiotherapy (RT) for more than a decade, which increased the efficacy of RT by precise interfractional patient localization.^1^, ^2^ In addition to the IGRT, adaptive RT (ART) has emerged as a new paradigm of RT. The ART aims to change and adapt the treatment to respond to additional information of patient change from the planning considering the current patient anatomy using additional imaging such as an on‐board imaging (OBI) or computed tomography (CT) scan.^3^, ^4^, ^5^ An OBI is often used in online ART to modify the contour and re‐optimize the plan with the patient on the couch, whereas an additional CT scan is required for an offline ART to re‐plan when an unexpected anatomical change occurs.

The image quality of the OBI system is a major component for the benefit of IGRT and ART. With a high‐quality OBI system, the patient can be more accurately aligned, and re‐optimization can more closely resemble the quality of a CT simulator in IGRT and ART. Among the OBI systems, CBCT attached to the linear accelerator (LINAC) gantry is the most widely used OBI system known to be highly effective for IGRT and online ART.

The Halcyon and Ethos treatment systems (Varian Medical Systems, Palo Alto, CA) are O‐ring LINAC unit with an integrated kilovoltage (kV)‐CBCT system and have become popular in the past several years. While these two systems possess identical hardware, with the difference being that only Ethos can perform online ART, the CBCT integrated into these systems showed a huge improvement over the conventional C‐arm LINAC CBCT systems in terms of larger field‐of‐view, longer scan range, faster scan time, and increasing signal‐to‐noise ratio (SNR).^1^, ^6^, ^7^

These O‐ring LINACs have recently been upgraded to version 4.0 with the latest version of the CBCT system, called HyperSight, which features an ultra‐fast scan and a larger detector with a high imaging performance. As HyperSight contains two modes for IGRT and CBCT‐based planning, it is expected that HyperSight will provide both rapid CBCT scans for more accurate patient motion management with IGRT mode and enhanced image quality for dose calculation of treatment planning with its CBCT for planning (CBCTp) mode. Since the performance of the OBI systems including CBCT is very important for IGRT and ART as previously mentioned,^8^, ^9^ the improvements in imaging performance have been of great interest.

In this perspective, we evaluated the image quality of HyperSight on quality assurance (QA) phantoms and compared it with its predecessor Halcyon 3.0 CBCT to characterize its imaging performance and set benchmark data for the routine imaging QA program. To do so, we aim to have a better understanding of the enhancement of HyperSight over Halcyon 3.0 CBCT and the strength of CBCTp mode over the IGRT mode.

## 2 MATERIAL AND METHODS

### 2.1 HyperSight CBCT imaging system

One of the primary enhancements of the HyperSight CBCT system lies in its detector technology. The HyperSight system incorporates a novel kV detector with an active area of 86 cm × 43 cm, twice the size of the Halcyon 3.0 CBCT detector. This large active area is positioned without lateral offset, enabling utilization of a full‐fan half‐arc (211‐degree) trajectory, thereby contributing to a mere 5.9 s (approximately 6 RPM) of image acquisition time compared to the half‐fan full‐arc of Halcyon 3.0 CBCT (16.6 s with fastest protocol).^10^ Employing Cesium Iodide (CsI) as the scintillator material, HyperSight achieves heightened x‐ray conversion efficiency, minimal lag, rapid readout, and superior radiation resistance compared to the Gadolinium Oxysulfide (GOS)‐based detector in the Halcyon 3.0 CBCT. Furthermore, HyperSight employs a larger kV tube with increased heat capacity (2.5 times higher) than the Halcyon 3.0 CBCT, a new kV collimator incorporating a movable bowtie filter, and a broader scan diameter (53.8 cm, extendable to 70 cm).^11^

These advancements are complemented by an advanced iterative CBCT reconstruction algorithm (iCBCT) called iCBCT Acuros (Acuros CTS‐based iCBCT), which features an efficient scattering removal with Acuros CTS and an advanced Monte‐Carlo based hardware scatter correction to generate high‐quality images, characterized by improved CT number accuracy and consistency.^12^, ^13^ A comparison between the Halcyon 3.0 CBCT system and the HyperSight is presented in Table 1.

**TABLE 1:** Comparison of the Halcyon 3.0 CBCT and the HyperSight (Halcyon 4.0 CBCT).

	Halcyon 3.0 CBCT	HyperSight (Halcyon 4.0 CBCT)
Detector active area	430 mm × 430 mm	860 mm × 430 mm
Panel type	a‐Si	a‐Si
Scintillator type	Gadolinium Oxysulfide (GOS)	Cesium Iodide (CsI)
Heat unit capacity	600 000 units	1 500 000 units
Anti‐scatter grid ratio	15:1	15:1
Lateral offset	17.5 cm (half‐fan)	0 cm (full‐fan)
Gantry speed	4 RPM	6 RPM
Scan time (minimum)	16.6 s	5.9 s
Scan diameter (maximum)	49.1 cm	53.8 cm
Source‐to‐imager distance	154 cm	154 cm
Source‐to‐axis distance	100 cm	100 cm
Reconstruction algorithms	FDK, iCBCT	FDK, iCBCT, iCBCT Acuros
kV collimator	fixed bowtie filter	movable bowtie filter

Abbreviations: CBCT, cone‐beam computational tomography; FDK, Feldkamp–Davis–Kress; kV, kilovoltage.

### 2.2 Imaging protocols of HyperSight CBCT imaging system

Table 2 shows preset imaging protocols available on the HyperSight system. Most of these protocols can be taken in either of the two modes: IGRT and CBCTp. The IGRT mode is designed for patient localization, while the CBCTp mode is more suitable for treatment planning. CBCTp mode provides high CT number accuracy without necessitating additional CT scans and typically comes with a high‐exposure setting (as high as Halcyon 3.0 CBCT), a larger slice thickness by default, and iCBCT Acuros reconstruction algorithm. We chose to use the vendor's default setting of the head, thorax, pelvis, and pelvis large protocols for both IGRT and CBCTp mode of HyperSight and for Halcyon 3.0 CBCT (detailed in Table 3). Feldkamp–Davis–Kress (FDK) and iCBCT were both used for Halcyon 3.0 CBCT and HyperSight IGRT mode, and iCBCT Acuros were used for HyperSight CBCTp mode.

**TABLE 2:** Available HyperSight default kV CBCT protocols.

Modes	Protocols	Energy [kV]	Exposure range [mAs]	Default exposure [mAs]	Default CTDIvol [mGy]	DLP [mGy^a^ cm]	Scan time [s]	Reconstruction diameter [cm]	Slice thickness [mm]
IGRT	Head^a^	100	46–169	88.00	2.03	30.5	5.9	28.2	2.0
	Thorax^a^	125	92–348	175.90	3.34	50.1	5.9	53.8	2.0
	Pelvis^a^	125	235–920	470.35	8.94	134.0	5.9	53.8	2.0
	Pelvis large^a^	140	266–869	527.71	13.56	203.4	5.9	53.8	2.0
	Image gently	80	34–101	52.80	0.58	8.7	5.9	28.2	2.0
	Image gently Large	100	34–110	58.98	1.36	20.4	5.9	38.4	2.0
	Head low dose	100	29–55	29.33	0.68	10.2	5.9	28.2	2.0
	Breast low dose	100	29–55	29.33	0.28	4.3	5.9	53.8	2.0
	Breast	125	29–55	29.33	0.56	8.4	5.9	53.8	2.0
CBCTp	Head^a^	125	210–1665	806.15	34.66	520.0	10.0	28.2	3.0
	Thorax^a^	125	184–971	703.61	13.37	200.5	5.9	53.8	3.0
	Pelvis^a^	125	133–971	527.71	10.03	150.4	5.9	53.8	3.0
	Pelvis large^a^	140	263–1490	1004.95	25.83	387.4	10.0	53.8	3.0
	Head pediatric	125	112–971	409.00	17.59	263.8	5.9	28.2	3.0
	Head and neck	125	184–971	703.61	30.26	453.8	5.9	28.2	3.0
	Body pediatric	125	41–573	146.59	2.79	41.8	5.9	53.8	3.0
	Breast	125	112–971	409.00	7.77	116.6	5.9	53.8	3.0
	Thorax slow	125	303–2774	703.16	13.36	200.4	58.6	53.8	3.0
	Abdomen	125	133–971	527.71	10.03	150.4	5.9	53.8	3.0
	Abdomen large	140	225–874	873.65	22.45	336.8	5.9	53.8	3.0

Abbreviations: CBCTp, CBCT‐planning mode; IGRT, image‐guided radiation therapy; kV, kilovoltage.

^a^ Protocol presets used in this study.

**TABLE 3:** Imaging protocols of Halcyon 3.0 CBCT system used in this study.

Protocols	Energy [kV]	Default exposure [mAs]	Default CTDIvol [mGy]	Scan time [s]	Reconstruction diameter [cm]	Slice thickness [mm]
Head	100	138.9	3.67	16.6	28.2	2.0
Thorax	125	300.62	6.01	30.8	49.2	2.0
Pelvis	125	1074	21.48	36.7	49.2	2.0
Pelvis large	140	1456.2	38.44	40.6	49.2	2.0

Abbreviations: CBCT, cone‐beam computational tomography; kV, kilovoltage.

### 2.3 Image quality comparison: Halcyon 3.0 CBCT versus HyperSight

Among several CBCT image quality tests suggested in the American Association of Physicists in Medicine task group report 179,^14^ we evaluated four metrics (spatial resolution, contrast resolution, uniformity, noise, and CT number linearity) using Catphan 604 (The Phantom Laboratory, Salem, NY) which is a common phantom used for routine imaging QAs. Catphan 604 comprises five modules, including slice geometry and sensitometry, bead geometry, high resolution, low contrast, and uniformity modules enclosed in a 20‐cm housing.^15^ CT scan images of each test were analyzed through the TotalQA (Image Owl, Inc., Greenwich, NY) software and in‐house Python codes to acquire measurements of each test. We also evaluated the CT number calibration error using a Gammex Advanced Electron Density phantom (Model 1467, Sun Nuclear, Melbourne, FL) with various tissue surrogate inserts.

### 2.4 Imaging performance characterization metrics

#### 2.4.1 Spatial resolution

To quantify spatial resolution, a fitted Modulation Transfer Function (MTF) curve was employed, derived through Fast Fourier Transform analysis of the Line Spread Function encompassing five key MTF points acquired from the high‐resolution module (CTP732) of Catphan 604 using the TotalQA software. The frequency (cycles/mm) at 50% of the MTF, known to be suitable for comparing the sharpness of imaging systems, along with 25%, 10%, and 5%, were used to measure the spatial resolution.^16^ The spatial resolution is proportional to the pixel density, so we also looked at the normalized frequency (cycle/pixel), which was obtained by dividing the frequency (cycle/mm) by the pixel density (pixel/mm).^17^

#### 2.4.2 Contrast resolution

To quantify the contrast resolution, we measured the contrast‐to‐noise ratio (CNR) and minimum detectable diameter of contrast rods.

First, we measured the CNR using polystyrene and low‐density polyethylene sensitometry plugs of the CTP732 module of Catphan 604 using TotalQA, calculated as follows.

$$CNR\;=\;\left(CT_{poly}-CT_{ldpe}\right)/\sqrt{\sigma_{poly}^{2}+\sigma_{ldpe}^{2}}$$

Further details regarding the region of interest (ROI) selection and CNR computation are elaborated in the subsequent CT number accuracy section. Since the standard deviation of the CT number is inversely proportional to the square root of the tube current (mAs),^19^ tube current‐normalized CNR was calculated by dividing the CNR and noise with the square root of the tube current ($CNR/\sqrt{mAs}$) to exclude the impact of the different tube currents of each protocol.^18^, ^19^, ^20^

In addition, to assess the nonlinear contrast resolution, we measured the minimum detectable diameter of contrast rods using the CTP730 low contrast module of the Catphan 604. This module contains nine rods with diameters of 1, 2, 3, 4, 5, 6, 7, 8, and 9 mm, in addition to 15 mm, for each contrast level: 1%, 0.5%, and 0.3%. The minimum detectable diameter of a contrast rod is defined as the smallest target diameter detectable for each of the three contrast values.

#### 2.4.3 Uniformity

With the ROIs and their mean CT numbers, the absolute deviation between the mean CT number ($m_{ROI}$) of the center ROI and upper, right, lower, and left ROIs were evaluated. The uniformity is calculated as the maximum of the absolute deviations of mean HU value of upper, right, lower, and left ROIs from the center ROI as follows.

$$uniformity\;=\;max\left(abs\left(mean_{center}-mean_{upper,\;right,\;lower,\;left}\right)\right)$$

ROIs of the upper, right, lower, left, and center regions of the Catphan 604 (CTP729 uniformity module) were selected with a diameter of 10% (i.e., 20.1 mm) of the phantom's diameter, following the guidance in International Electrotechnical Commission (IEC) 61223. The peripheral uniformity ROIs (upper/right/lower/left regions) were positioned so that the outer edge of the ROI lies 10 mm from the border of the uniformity module, and the central ROI is centered on the phantom.

#### 2.4.4 Noise

The noise was quantified with the standard deviation of the CT number in the center ROI of the Catphan uniformity module as follows.

$$Noise\;=\sigma_{center\;ROI}$$

Similar to the uniformity, ROIs of the center region of the Catphan 604 (CTP729 uniformity module) were selected to be 40% (i.e., 80.4 mm) of the phantom's diameter from the TotalQA software, following the guidance in IEC 61223. We also evaluated the normalized noise with the square root of the tube current (mAs).

#### 2.4.5 CT number linearity and calibration error

A linear regression analysis was performed between the linear attenuation coefficients and the average CT numbers to quantify the CT number linearity. Root‐mean‐squared error (RMSE) was calculated between the linear fit and the average CT number of each material in the Catphan sensitometry block. A 10‐mm diameter ROI was drawn for each material block of the CTP732 sensitometry module in Catphan 604. An average CT number of each material and effective energy were obtained.

Regarding CT calibration error, we measured the CT number accuracy of the HyperSight CBCTp mode and compared it to the HyperSight IGRT mode and a CT scanner SOMATOM go.Open Pro (Siemens Healthineers, Erlangen, Germany). To measure the CT number calibration error, we scanned the electron density phantom (Sun Nuclear, Melbourne, FL) containing 11 inserts with a wide range of electron density and obtained a calibration curve for HyperSight CBCTp mode and the CT scanner. The scanned inserts were LN‐300 and 450 Lung, General Adipose, Breast 50:50, True water, Brain, Liver, Blood 100, Inner bone, 50% CaCO_3_, Cortical bone in the head and body size configuration. We manually drew a circular ROI of 15 mm in diameter at the center of each insert in the scanned image and extracted the average CT number. Average CT numbers of the head and body phantom and the vendor‐provided electron density table were used to establish the CT number to electron density calibration curve. Calibration error was evaluated by calculating the relative error between the electron density table and the mapped electron density from the CT number of each head and body phantom.

## 3 RESULTS

**FIGURE 1:**
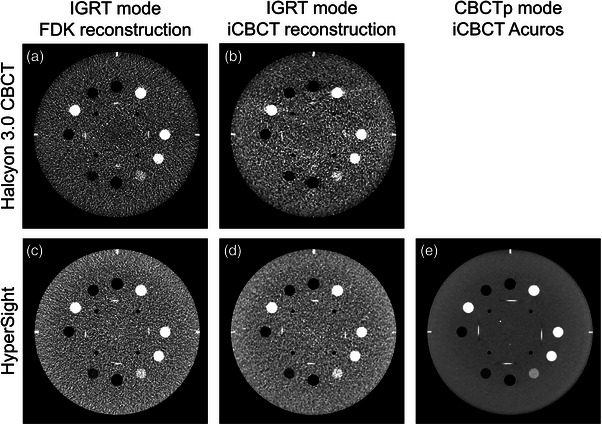
A representative CBCT image for each system, reconstruction algorithm, and mode. (a) Halcyon 3.0 CBCT IGRT mode with FDK reconstruction. (b) Halcyon 3.0 CBCT IGRT mode with iCBCT reconstruction. (c) HyperSight IGRT mode with FDK reconstruction. (d) HyperSight IGRT mode with iCBCT reconstruction. (e) HyperSight CBCTp mode with iCBCT Acuros reconstruction. CBCT, cone‐beam computational tomography; CBCTp, CBCT‐planning mode; FDK, Feldkamp–Davis–Kress; IGRT, image‐guided radiation therapy.

A representative CBCT image of Catphan 604 for each system (Halcyon 3.0 CBCT and HyperSight), algorithm (FDK, iCBCT, and iCBCT Acuros), and/or mode (IGRT and CBCTp mode) are shown in Figure 1. The detailed result of individual scan is shown in Table S1.

### 3.1 Spatial resolution

**FIGURE 2:**
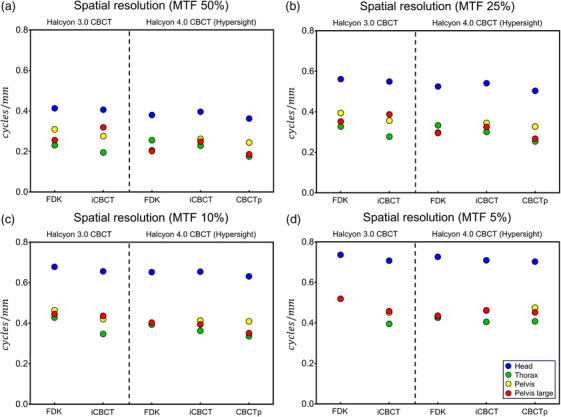
Comparison of spatial resolution (cycles/mm) as a frequency at MTF (a) 50%, (b) 25%, (c) 10%, and (d) 5% calculated from the Catphan 604 high‐resolution module. MTF, modulation transfer function.

**FIGURE 3:**
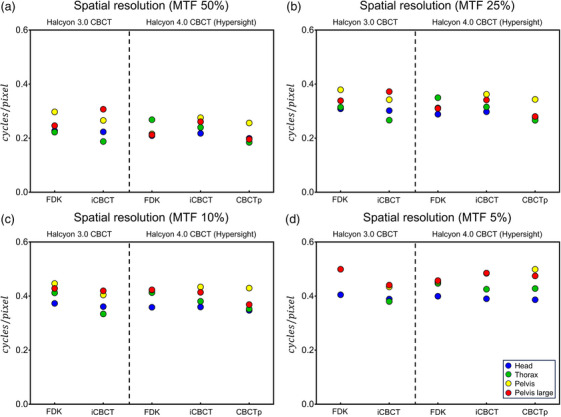
Comparison of spatial resolution (cycles/pixel) as a frequency at MTF (a) 50%, (b) 25%, (c) 10%, and (d) 5% calculated from the Catphan 604 high‐resolution module. MTF, modulation transfer function.

The spatial resolution (frequency at MTF 50%, 25%, 10%, and 5%) of 4 protocols (Head, Thorax, Pelvis, Pelvis large) are shown in Figure 2 (cycles/mm) and Figure 3 (cycles/pixel). HyperSight (both IGRT and CBCTp mode) showed similar spatial resolution to Halcyon 3.0 CBCT in both cycles/mm and cycles/pixel. The Head protocol showed the highest spatial resolution in Figure 2, but this was due to the small pixel size of the Head protocol. Most protocols exhibited similar range of spatial resolution in pixel‐size normalized result (cycles/pixel) in Figure 3.

### 3.2 Contrast resolution

**FIGURE 4:**
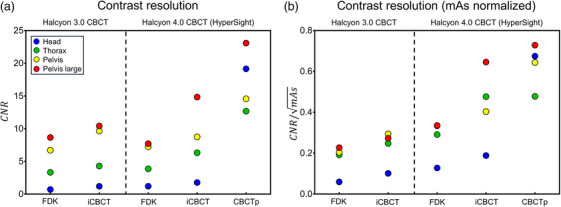
Comparison of contrast resolution as a CNR calculated from the Catphan 604 low contrast module. (a) CNR and (b) $CNR/\sqrt{mAs}$. Error bars (mean and standard deviation) are from the results of the head, thorax, pelvis, and pelvis large protocols. CNR, contrast‐to‐noise ratio.

The raw and tube current‐normalized CNR are shown in Figure 4, and the minimum detectable diameter of a contrast rod is shown in Table 4.

**TABLE 4:** Smallest detectable diameter of contrast rods at each mode, contrast level, and protocol.

Imager	Mode	1% contrast				0.5% contrast				0.3% contrast			
		Head	Thorax	Pelvis	Pelvis large	Head	Thorax	Pelvis	Pelvis large	Head	Thorax	Pelvis	Pelvis large
Halcyon 3.0 CBCT	IGRT(FDK)	9	5	3	3	N/D	9	6	5	N/D	15	9	9
	IGRT(iCBCT)	15	9	5	4	N/D	N/D	N/D	N/D	N/D	N/D	N/D	N/D
HyperSight	IGRT(FDK)	8	5	3	3	15	8	6	5	N/D	9	9	8
	IGRT(iCBCT)	8	5	3	2	15	9	7	6	N/D	N/D	N/D	15
	CBCTp	2	2	2	2	5	5	4	3	15	15	9	9

Abbreviations: CBCT, cone‐beam computational tomography; CBCTp, CBCT‐planning mode; FDK, Feldkamp–Davis–Kress; IGRT, image‐guided radiation therapy; N/D, none detected.

HyperSight IGRT mode showed higher CNR than Halcyon 3.0 CBCT even with almost half of the tube current (average tube current of the four protocols are 315 mAs in HyperSight and 742 mAs in Halcyon 3.0 CBCT). With the $CNR/\sqrt{mAs}$, HyperSight showed considerably higher contrast resolution than Halcyon 3.0 CBCT. HyperSight CBCTp mode had even higher CNR and $CNR/\sqrt{mAs}$ than the IGRT mode.

Though the iCBCT had better performance than the FDK for both Halcoyo30 CBCT and the HyperSight in CNR analysis, the nonlinear contrast analysis using minimum detectable diameter of a contrast rod has been shown that the FDK reconstruction algorithm performed better than the iCBCT reconstruction algorithm, especially at low contrast levels.

### 3.3 Uniformity

**FIGURE 5:**
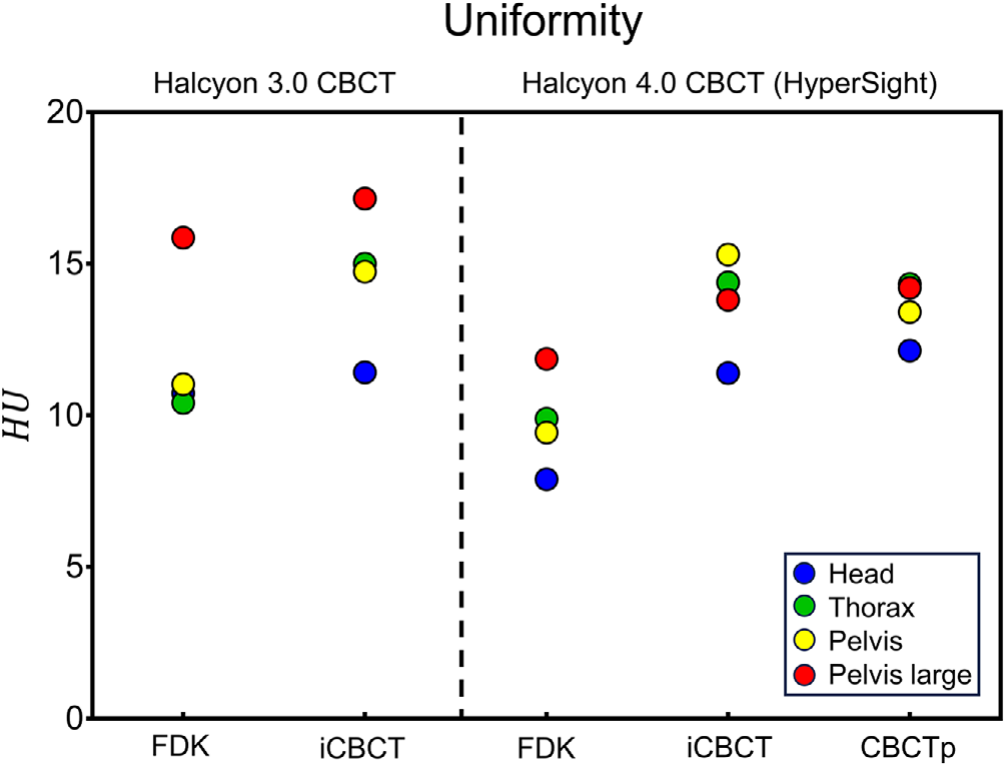
Comparison of uniformity as a MAD between the mean CT number of upper/lower/right/left and center ROI of the Catphan 604 uniformity module. Error bars (mean and standard deviation) are from the results of the head, thorax, pelvis, and pelvis large protocols. CT, computed tomography; MAD, maximum absolute deviation; ROI, region of interest.

The CT number uniformity of each system is presented in Figure 5. HyperSight IGRT mode showed marginally lower CT number deviation than Halcyon 3.0 CBCT for both FDK and iCBCT. FDK reconstruction algorithm showed lower CT number deviation (higher uniformity) compared to iterative algorithms for both Halcyon 3.0 CBCT and HyperSight. HyperSight CBCTp mode showed a CT number deviation similar to the IGRT mode.

### 3.4 Noise

**FIGURE 6:**
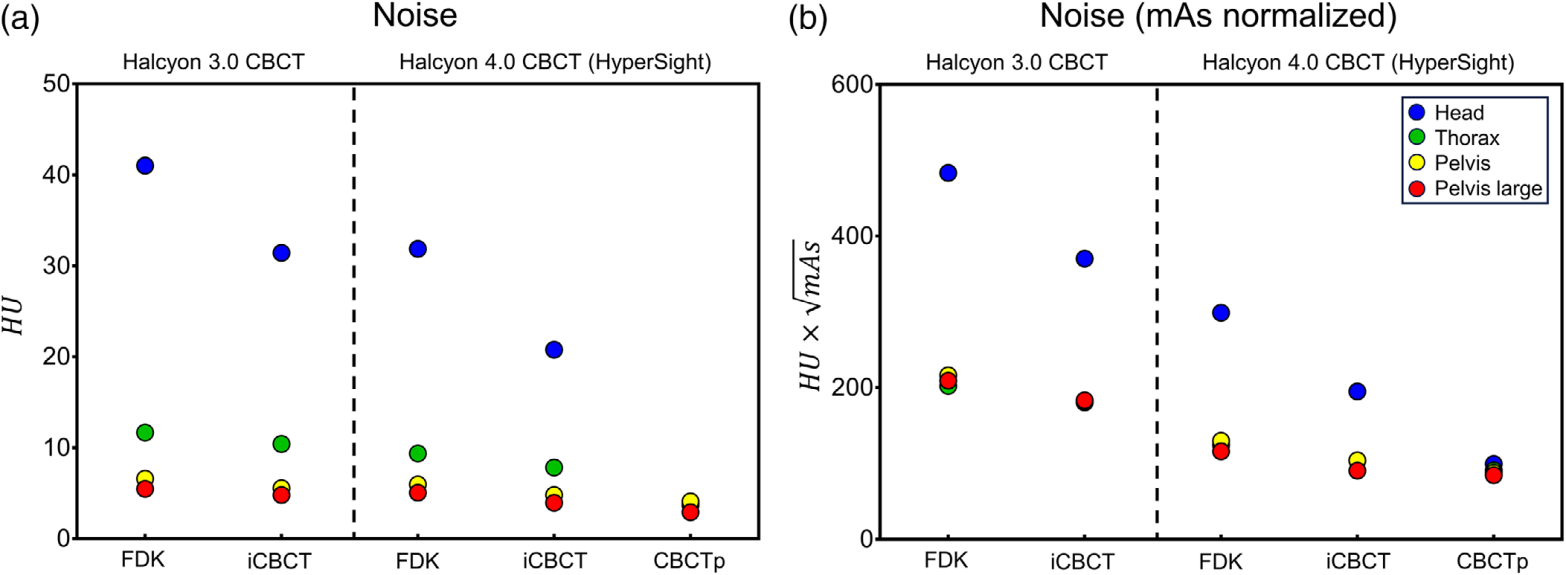
Comparison of noise as a standard deviation of CT number within the diameter 40% of the center ROI of the Catphan 604 uniformity module; (a) raw CT number noise (HU) and (b) tube current (mAs) normalized CT number noise in $HU\times \sqrt{mAs}$. Error bars (mean and standard deviation) are from the results of the head, thorax, pelvis, and pelvis large protocols. CT, computed tomography; ROI, region of interest.

The raw and tube current‐normalized noise are shown in Figure 6. HyperSight IGRT mode showed lower noise than Halcyon 3.0 CBCT, even with a lower tube current setting. With the mAs‐normalized noise ($noise\times \sqrt{mAs}$), HyperSight showed considerably lower noise than Halcyon 3.0 CBCT. HyperSight CBCTp mode had even lower noise than the IGRT mode in both noise and $noise\times \sqrt{mAs}$).

### 3.5 CT number linearity and calibration error

**FIGURE 7:**
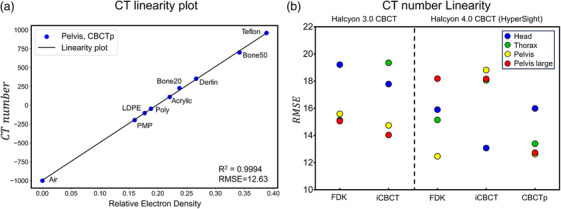
(a) CT number and linearity plot of HyperSight CBCTp mode pelvis protocol. (b) Comparison of CT number linearity represented by RMSE of CT linearity plot calculated from a ROI of the Catphan 604 sensitometry module. Error bars (mean and standard deviation) are from the results of the head, thorax, pelvis, and pelvis large protocols. CBCTp, CBCT‐planning mode; CT, computed tomography; RMSE, Root‐mean‐squared error; ROI, region of interest.

The CT number linearity is shown in Figure 7. The RMSE of the CT number linearity curve was similar between Halcyon 3.0 CBCT and HyperSight. For HyperSight, the IGRT mode with the FDK reconstruction algorithm showed lower RMSE than the iCBCT reconstruction, and the CBCTp mode showed the lowest RMSE. In the case of the R^2^ of the linearity plot, Halcyon 3.0 CBCT (0.9991 for FDK and 0.9990 for iCBCT) and HyperSight (0.9991 for FDK and 0.9990 for iCBCT) were the same, while the HyperSight CBCTp mode showed the highest, 0.9993.

**FIGURE 8:**
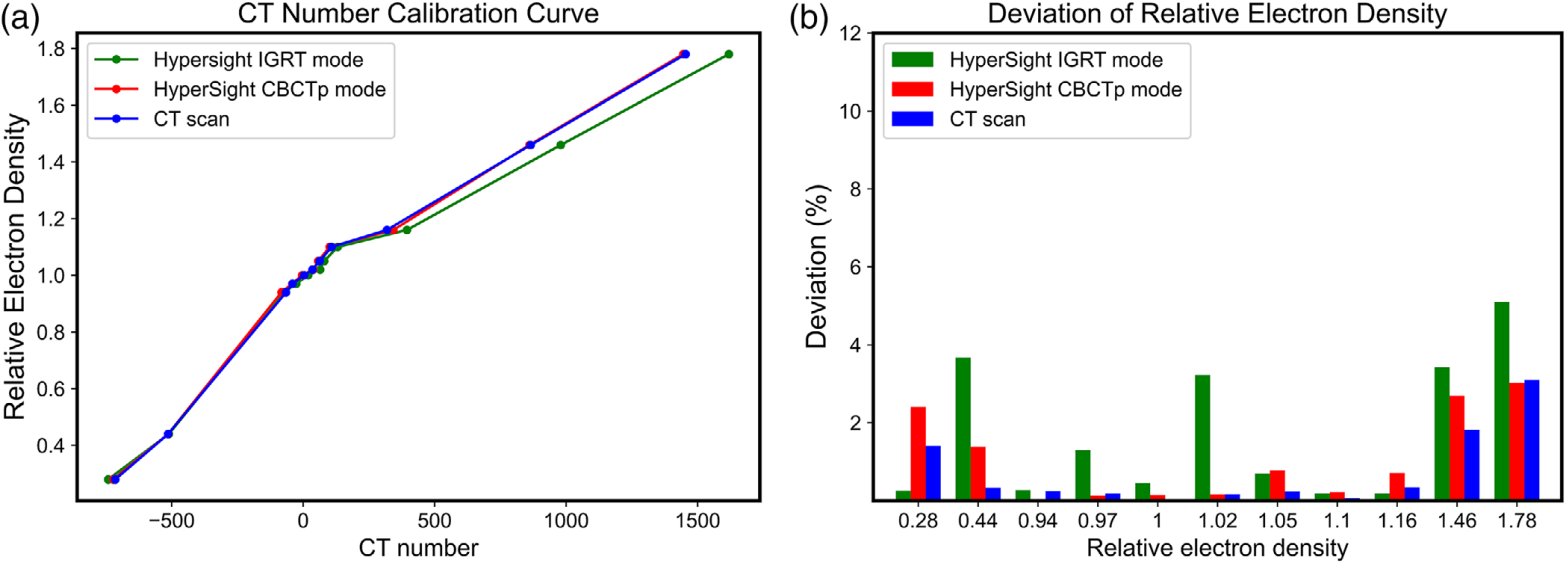
Comparison of (a) CT number calibration curve of HyperSight CBCTp mode and full CT scan obtained using electron density phantom and (b) relative deviation of relative electron density. CBCTp, CBCT‐planning mode; CT, computed tomography.

The CT number calibration curve of the full CT scanner and the HyperSight CBCTp mode are shown in Figure 8A, and the relative deviations of the three scans (HyperSight IGRT mode, CBCTp mode, and full CT scan) are shown in Figure 8B. The HyperSight CBCTp mode exhibited a CT number accuracy (0% means 100% accurate) of 1.06%, while the full CT scan showed 0.72%, and the HyperSight IGRT mode showed 1.71%. The relative deviations of the three scans in each insert were large in material with relative electron density greater (bone) or less than 1 (lung) and small in material near relative electron density of 1 (soft tissues).

## 4 DISCUSSION

In this study, we conducted a comprehensive image quality characterization of the HyperSight CBCT system and compared it with the existing Halcyon 3.0 CBCT system. Briefly, HyperSight achieved considerably improved CNR and SNR, slightly higher uniformity, and similar spatial resolution compared to the Halcyon 3.0 CBCT for both IGRT and CBCTp mode. Regarding CT number accuracy, the CBCTp mode provided a CT number calibration error comparable to a CT simulator.

In terms of the image quality, the significant improvement of HyperSight IGRT mode in CNR and SNR is putatively due to the thick CsI scintillator (more than 2 times thicker than the Halcyon 3.0 CBCT GOS scintillator) and reduced grid‐to‐scintillator distance (less than one‐third of Halcyon 3.0 CBCT). The thicker scintillator produces more photons when interacting with input radiation, and the reduced grid‐to‐scintillator distance prevents the system from losing photons produced by the scintillator, both leading to a higher detector efficiency. The HyperSight CBCTp mode showed even higher CNR and SNR with similar uniformity and spatial resolution than the IGRT mode, and these differences are likely owing to the reconstruction algorithm (iCBCT Acuros), which would enhance the overall image uniformity and CNR/SNR by suppressing noise effectively.

In the case of CT number linearity and accuracy, CBCTp mode with iCBCT Acuros reconstruction showed a closely matching CT number calibration error (1.06%) compared to the CT scanner (0.72%). As shown in Figure 8, CBCTp mode exhibited a very close CT number to that of the CT scanner between the head and the body phantom, and the calibration error mainly came from non‐soft tissue materials, such as lung and bone. This high CT number accuracy of CBCTp mode would be attributed to the hardware improvements (larger detector and slice thickness) and advanced scatter correction of the iCBCT Acuros reconstruction algorithm, improving overall image quality and CT number accuracy.^13^ Note that the CT number of HyperSight saturates at 7000 HU, so a metal implant such as titanium or stainless steel will need to be identified, contoured, and overridden with the correct material. As the CBCTp mode of HyperSight received the Food and Drug Administration (FDA) 510(k) clearance in fall 2023, Halcyon equipped with HyperSight could utilize the CBCTp mode as an alternative to a CT scanner for initial or adaptive planning.

The ultra‐fast scan time (6–10 s) is undoubtedly a key feature of HyperSight, on top of the enhancement in image quality. An ultra‐fast scan of HyperSight can dramatically reduce patient movement and corresponding motion artifacts during the scan. It is foreseeable that there will be a greater difference in image quality between HyperSight and other systems if the evaluation is done on a moving target, not static phantoms.^21^ Moreover, breath‐hold scans can be more easily utilized because the scan can be finished within a single breath‐hold, which not only reduces the motion artifacts but also is easy on the patient and enables faster treatment.^22^

In our experience, the x‐ray tube with a larger capacity of HyperSight also demonstrated its clinical usefulness. The heat unit capacity of the HyperSight (more than twice that of the Halcyon 3.0 CBCT) makes the HyperSight less prone to tube overheating, which enables taking several consecutive high mAs scans before waiting for the tube to cool down. Halcyon 3.0 CBCT, on the other hand, cannot take two large pelvis scans in a row, which may be an obstacle for an extended CBCT scan.

While our study provides insights into the imaging performance of the HyperSight CBCT system, several limitations should be acknowledged. First, our findings are based solely on static Catphan data measurements and they may not reflect the performance of HyperSight in clinical scenarios but are aimed at gauging the overall performance of HyperSight and providing benchmark data for routine QA. Moreover, since HyperSight is a cone‐beam system, placement of the phantom may also affect the image quality due to different scattering path of the kV beam. In this study, the isocenter was placed at the center of the four modules. Therefore, our findings should be interpreted with an emphasis on the observed tendencies rather than the specific values themselves. Second, our assessment primarily focuses on short‐term image quality metrics using standardized phantoms. Long‐term image evaluation with system performance change over time is not addressed in this study. Third, this study is based on data from a single institution. Therefore, additional research by other institutions that have implemented HyperSight would be needed to further validate our results and provide a clearer understanding of the performance of the HyperSight CBCT system.

## 5 CONCLUSION

We thoroughly examined the image quality of the HyperSight CBCT imaging system (i.e., Halcyon 4.0) in terms of spatial resolution, contrast resolution, uniformity, noise, and CT number accuracy. HyperSight is a highly advanced CBCT system that enables ultra‐fast scans with improved image quality compared to Halcyon 3.0 CBCT and CT number accuracy close to a CT scanner. It is expected that the scan speed and image quality of HyperSight would bring huge benefits to enhance the quality and efficiency of the treatment, especially for patients requiring thoracic scans with breath‐hold.

## AUTHOR CONTRIBUTIONS

Euidam Kim took the lead on data collection and analysis. Yang Kyun Park, Tianyu Zhao, and Yoonsun Chung provided guidance on the CBCT analysis and the use of TotalQA software. Eric Laugeman and Xiaodong Neo Zhao and Lee set up the experiment and collected data. All authors participated in the manuscript writing and revision.

## CONFLICT OF INTEREST STATEMENT

The authors declare no conflicts of interest.

## Supporting information

Supporting Information
